# The Spanish Version of the Internet Gaming Disorder Scale-Short Form (IGDS9-SF): Further Examination Using Item Response Theory

**DOI:** 10.3390/ijerph17197111

**Published:** 2020-09-28

**Authors:** Laura Maldonado-Murciano, Halley M. Pontes, Mark D. Griffiths, Maite Barrios, Juana Gómez-Benito, Georgina Guilera

**Affiliations:** 1 Faculty of Psychology, University of Barcelona, Passeig de la Vall d’Hebron, 171, 08035 Barcelona, Spain; mbarrios@ub.edu (M.B.); juanagomez@ub.edu (J.G.-B.); 2 Institute of Neurosciences, University of Barcelona, Passeig de la Vall d’Hebron, 171, 08035 Barcelona, Spain; 3 School of Psychological Sciences, University of Tasmania, Newnham Campus, Building O, Launceston, TAS 7250, Australia; contactme@halleypontes.com; 4 The International Cyberpsychology and Addictions Research Laboratory (iCARL), University of Tasmania, Launceston, TAS 7249, Australia; 5 International Gaming Research Unit, Psychology Department, Nottingham Trent University, Nottingham NG1 4FQ, UK; mark.griffiths@ntu.ac.uk

**Keywords:** gaming addiction, internet gaming disorder, Internet Gaming Disorder Scale-Short Form, validation, item response theory

## Abstract

Internet gaming disorder (IGD) has been recognized by the American Psychiatric Association (APA) as a tentative disorder in the latest (fifth) revision of the *Diagnostic and Statistical Manual of Mental Disorders* (DSM-5). However, psychometric evaluation of the nine IGD criteria remains necessary to further enhance its assessment. Therefore, the objective of this study was to evaluate the psychometric properties of the Spanish version of the Internet Gaming Disorder Scale-Short Form (IGDS9-SF). The internal structure, internal consistency, temporal stability, and relationships with other variables were assessed. Furthermore, a polytomous item response theory (IRT) approach was used to evaluate the performance of each item and the test as a whole. A sample of 388 online gamers (53.61% women, mean age 25.45 years, standard deviation (SD) = 9.62) was recruited for this study. Similar to previous research, the results supported a one-factor structure for the IGDS9-SF, adequate internal consistency and temporal stability of scores, goodness of fit of the items to the graded response model (GRM), and more precise scores at high trait levels to assess IGD in Spanish populations. These findings corroborate the suitability of the Spanish IGDS9-SF for clinical assessment and research within Spanish-speaking populations.

## 1. Introduction

Internet gaming disorder (IGD) has been recognized by the American Psychiatric Association (APA) in May 2013 as a tentative disorder in the latest (fifth) revision of the *Diagnostic and Statistical Manual of Mental Disorders* (DSM-5) [1]. The APA defined IGD as “persistent and recurrent use of the Internet to engage in games, often with other players, leading to clinically significant impairment or distress” [1] (pp. 795). To be diagnosed with IGD, five or more of the following nine diagnostic criteria must be endorsed over a period of 12 months: (i) preoccupation with internet gaming; (ii) withdrawal symptoms when internet gaming is not possible (e.g., irritability, anxiety); (iii) tolerance, resulting in the need to spend increasing amounts of time engaged playing internet games; (iv) unsuccessful attempts to control the participation in internet gaming; (v) loss of interests in previous hobbies and entertainment as a result of, and with the exception of, internet gaming; (vi) continued excessive use of internet games despite knowledge of psychosocial problems; (vii) deceiving family members, therapists, or others regarding the amount of internet gaming; (viii) use of internet gaming to escape or relieve a negative mood (e.g., feelings of helplessness, guilt); and (ix) jeopardizing or losing a significant relationship, job, or educational or career opportunity because of internet gaming.

In terms of the extent of problems caused by IGD, previous research suggested that prevalence rates of IGD tend to differ significantly from study to study [2]. The disparities in IGD prevalence rates reported across studies are partly due to discrepancies in the assessment framework and approach adopted by researchers when assessing the construct [3,4]. A recent meta-analysis [5] further corroborated the issue of heterogeneity in IGD prevalence rates, noting that studies often report inconsistent prevalence estimates (ranging from 0.6% [6] to 19.9% [7]). Nevertheless, the results of this meta-analytic study suggested a mean prevalence rate of IGD of 4.6% among adolescents, with males presenting higher prevalence estimates than females (6.8% vs. 1.3%, respectively).

Although there is evidence supporting the therapeutic and health-related benefits of judicious gaming among adolescents and adults [8], excessive and disordered gaming can negatively affect gamers’ psychological, social, and physical health [9], further leading to the experience of functional impairments [10], chronic stress [11], and sleep disturbances [12]. With regards to cross-sectional associations, previous research has found several factors to be associated with IGD, including, but not limited to increased levels of anxiety, depression, stress [11,13,14,15,16,17,18], and impulsivity [19,20]. Additional personality traits have been associated with IGD, such as high levels of neuroticism and low levels of conscientiousness [21,22,23,24].

The preliminary inclusion of IGD in the DSM-5, and subsequent official recognition of gaming disorder as a mental health disorder in the eleventh revision of the *International Classification of Diseases* (ICD-11) [25], has further highlighted the need for researchers to (i) gather additional empirical evidence to identify the defining features of disordered gaming, (ii) obtain cross-cultural data on the utility of the official diagnostic criteria, (iii) accurately determine its prevalence rates among representative samples in different countries around the world, and (iv) examine its correlates and associated biological and psychological features [4,26,27,28].

Over the past few years, researchers have focused on improving the clinical and psychometric assessment of IGD through the development of several standardized psychometric tools assessing IGD according to the nine official criteria proposed by the APA in the DSM-5 [1]. Although there are several psychometrically robust assessment tools to evaluate IGD based on the nine diagnostic criteria developed by the APA (see Bernaldo-De-Quirós, Labrador-Méndez, Sánchez-Iglesias, and Labrador [29], and Kuss and Pontes [30]), the Internet Gaming Disorder Scale-Short Form (IGDS9-SF) [4] has thus far been one of the most widely used tools internationally to assess IGD [29] due to the exceptional coverage of the DSM-5 diagnostic framework, its brevity, ease of scoring, excellent psychometric properties, and extensive data, demonstrating its utility in a wide range of countries and cultures [31,32].

The IGDS9-SF was originally developed in English and subsequently psychometrically validated and adapted to various languages, including Albanian [33], Chinese [34], Czech [35], German [36], Italian [37], Malay [38], Persian [31], Polish [39], European and South American Portuguese [15,40], Slovenian [41], Spanish [42], and Turkish [32,43]. Furthermore, its cross-cultural validity has been further supported by several international studies examining its measurement invariance (i.e., configural, metric, and scalar) across different countries [33,44,45]. Taken together, these studies highlight the suitability of the IGDS9-SF in assessing IGD.

Given that there are currently 15 million gamers in Spain aged between 6- and 64-years-old [46], it is paramount to have valid and reliable psychometric tools developed for the Spanish context that assess IGD under a common and internationally accepted framework such as the APA diagnostic framework and its nine IGD DSM-5 criteria. In a recent study, Beranuy and colleagues [42] developed and validated a Spanish version of the IGDS9-SF in a sample of young students and further examined its psychometric properties utilizing a classical test theory (CTT) framework (e.g., performing confirmatory factor analysis [CFA]). The authors reported that the Spanish IGDS9-SF was a valid and reliable tool and provided further evidence of its unidimensionality; adequate internal consistency; configural and metric gender invariance; and configural, metric, and scalar invariance across age (≤17 years vs. ≥18 years), alongside evidence of convergent validity.

Notwithstanding this, there is still a need to further examine the psychometric properties of the IGDS9-SF in terms of its temporal stability, item performance, and measurement precision. Additionally, the investigation of how IGDS9-SF scores relate to other measures of interest (e.g., personality, depression, anxiety, and stress) needs to be further scrutinized in culturally-diverse samples. Therefore, the present study aims to further contribute to the psychometric investigation of the IGDS9-SF by examining its utility among a wider Spanish community sample, while providing new data regarding its validity and reliability from the point of view of CTT and item response theory (IRT).

## 2. Materials and Methods

### 2.1. Participants and Procedures

In order to take part in the study, participants had to meet the following inclusion criteria: (i) be at least 16 years old and (ii) had played video games at least once in their lifetime. The study was conducted in accordance with the Declaration of Helsinki and approved by the Committee on Bioethics of the University of Barcelona (IRB00003099). Online informed consent was obtained from all participants. The online survey was developed and hosted on *Qualtrics* and included questions assessing participants’ gaming behaviors, personality traits, and psychiatric symptoms. The online survey was advertised between 24 March and 18 June 2019 on different social media platforms, online forums (e.g., video game and parental care forums), posters placed in different places in Barcelona (Spain), and in the online university virtual campus of the University of Barcelona.

A total sample of 468 participants completed the online survey. Of these, 73 were excluded because of having not completed the entire survey, and another seven were further excluded because they indicated they had never played video games. Consequently, a total of 388 participants were finally included in the study’s statistical analyses. In terms of the main sociodemographic characteristics, 53.61% of all participants were female and the mean age of the sample was 25.45 years (SD = 9.62; range: 16–72 years). Most participants indicated they had completed a higher education degree (39.43%) or finished secondary education (38.14%). With regards to gaming behaviors, participants played on average 1.42 h per day on working days (SD = 1.86; range: 0–15 h) and 2.79 h per day on non-working days (SD = 2.71; range: 0–16 h).

In order to test the temporal stability of the IGDS9-SF, a subsample of 31 participants agreed to participate in a second test administration that took place one month after the initial data collection.

### 2.2. Instruments

*Sociodemographic data:* The survey collected data on participants’ gender, age, educational level achieved, and gaming-related behaviors (e.g., time spent gaming during the working days and non-working days such as weekends and holidays).

*Internet Gaming Disorder Scale-Short Form* (IGDS9-SF) [4]: The IGDS9-SF was used to assess IGD. The IGDS9-SF includes nine items reflecting the nine IGD criteria proposed by the APA in the DSM-5 criteria. All nine items are scored on a five-point Likert scale ranging from 1 (*never*) to 5 (*very often*), with higher scores indicating higher levels of disordered gaming symptoms. More recently, a study using a clinical sample has suggested a cut-off of 32 to distinguish between disordered and non-disordered gamers [47]. The IGDS9-SF has been previously validated in several languages, and all previous studies have confirmed its unidimensionality and sound psychometric properties. Given that, at the time of conducting the present study, no Spanish version of the IGDS9-SF had been developed, parallel translation and reconciliation procedures for the translation of the IGDS9-SF from English into Spanish were conducted. Firstly, two psychologists independently translated the original scale from English into Spanish. Secondly, a third independent translator identified and resolved any discrepancies between the alternative Spanish forward translations [48]. The final version of the scale used in the present study is shown in Appendix A, Table A1. Discrepancies between this translation and Beranuy et al.’s [42] version were minimal, and both retained the core psychological meaning of the original English version. The Cronbach’s alpha and the Omega coefficients for the IGDS9-SF in the present study were α = 0.90 and ω = 0.84, respectively.

*Internet Gaming Disorder Test-20* (IGD-20 Test) [4]: The IGD-20 Test is a standardized tool developed to assess the severity of IGD using the nine criteria for IGD in the DSM-5. The IGD-20 Test includes a set of 20 items assessing the detrimental effects of IGD by examining both online and/or offline gaming activities occurring over a 12-month period. All 20 items are responded to using a five-point Likert scale ranging from 1 (*strongly disagree*) to 5 (*strongly agree*). The Spanish IGD-20 Test has been previously shown to exhibit excellent psychometric properties in terms of factorial structure, internal consistency, and criterion-related validity [49]. The Cronbach’s alpha and the Omega coefficients for the IGD-20 Test in the present study were α = 0.95 and ω = 0.77, respectively (see Table 1).

*Mini International Personality Item Pool-Five-Factor Model-Positively Worded* (Mini-IPIP-PW) [50]: The Mini-IPIP-PW comprises 20 items answered using a five-point Likert scale response from 1 (*totally disagree*) to 5 (*totally agree*). The Mini-IPIP-PW evaluates the following personality traits based on the five factor model of personality [51]: extraversion (E), agreeableness (A), conscientiousness (C), neuroticism (N), and openness (O). The version used in the present study is the positively worded version, which differs from the original Mini-IPIP in that all the items are positively worded. The Spanish Mini-IPIP-PW has shown high levels of reliability and convergent and predictive validity [52]. The Cronbach’s alpha and the Omega coefficients for the Spanish Mini-IPIP-PW in the present study were all adequate, with the exception of factors E and N (see Table 1).

*Depression, Anxiety and Stress Scales (DASS-21)* [53]*:* The DASS-21 was used to assess psychiatric symptoms of depression, anxiety, and stress. The DASS-21 includes 21 items that can be responded to on a four-point Likert scale from 0 (*did not apply to me at all*) to 3 (*applied to me very much, or most of the time*). The Spanish DASS-21 has been shown to exhibit adequate internal consistency, satisfactory convergent validity, and acceptable discriminant validity [54]. The Cronbach’s alpha and the Omega coefficients for the Spanish DASS-21 in the present study were all adequate, with the exception of stress (see Table 1).

### 2.3. Data Analysis

The unidimensionality of the IGDS9-SF was assessed using CFA with the diagonal weighted least square (DWLS) estimator, because the items have a Likert-point scale, which can be considered as ordinal. The model fit of the measurement model was assessed with the comparative fit index (CFI), the Tucker–Lewis index (TLI), the root mean square error of approximation (RMSEA), and the standardized root mean square residual (SRMR). Goodness of fit was interpreted following the recommended guidelines proposed by Hu and Bentler [55], suggesting values of CFI ≥ 0.95, TLI ≥ 0.95, RMSEA ≤ 0.06, and SRMR ≤ 0.08 as adequate fit.

Moreover, convergent validity was assessed with the average variance extracted (AVE) coefficient for the IGD latent factor. Construct reliability included an examination of several reliability coefficients (i.e., Cronbach’s alpha and temporal stability, composite reliability [CR], and Omega coefficient [ω]), because the Cronbach’s alpha coefficient presents with several limitations (e.g., the assumptions of uncorrelated errors, tau-equivalence, and normality) [56]. The relationship between IGD as assessed by the IGDS9-SF and the other variables was assessed by computing Pearson correlation coefficients.

In order to further complement the aforementioned CTT analyses, a follow-up IRT analysis for polytomous items was conducted on the nine IGDS9-SF items. In comparison with CTT, IRT models present with unique advantages such as giving more information about the quality of the items and providing measures of precision at different levels of the trait (*θ*) [57]. Because of the ordinal nature of the nine IGDS9-SF items, three models for polytomous items were fitted [58]. These were the partial credit model (PCM) [59], the generalized partial credit model (GPCM) [60], and the graded response model (GRM) [61]. Their fits were compared in terms of the Akaike information criteria (AIC) and the Bayesian information criteria (BIC), selecting the model with lower values because they indicate a closer fit to the true model [62]. Under the GRM, which was the model that showed a better fit to the data, in a polytomous item *i* with *X* ordered response categories, the probability of obtaining *X_i_* points or higher (*X_i_* = 0, 1, …, *m_i_*) can be expressed as follows:

(1) $$P^{*}\left(X_{i}|\theta ,a_{i},\;\delta_{X_{i}}\right)=\frac{e^{a_{i}\left(\theta -\delta_{X_{i}}\right)}}{1+e^{a_{i}\left(\theta -\delta_{X_{i}}\right)}}$$

where θ is the latent trait, $a_{i}$ is the discrimination parameter for item *i*, $\delta_{X_{i}}$ is the category boundary location for the category *X_i_*, and $P^{*}\left(X_{i}|\theta ,a_{i},\;\delta_{X_{i}}\right)$ is the probability of a person obtaining a score of *X_i_* or higher.

The IRT results also provided the item fit parameters using the S-χ^2^ statistic, the item characteristic curves, and the information function of both items and the scale as a whole including all nine items of the IGDS9-SF. The statistical analyses were carried out with the R statistical package (R Foundation for Statistical Computing, Vienna, Austria) [63], using the packages *lavaan* [64] for the CFA, and *semTools* [65] and *mirt* [66] for the IRT analysis.

## 3. Results

### 3.1. Dimensionality

**Figure 1:**
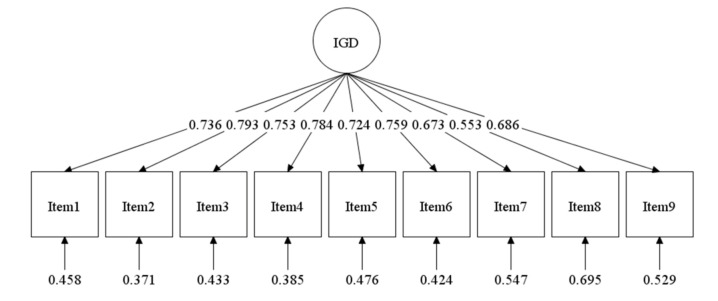
Path diagram with summary of the confirmatory factor analysis (CFA) obtained from the nine items of the Internet Gaming Disorder Scale-Short Form (IGDS9-SF). IGD: internet gaming disorder.

In order to investigate the dimensionality of the Spanish IGDS9-SF, CFA on the nine items was carried out. The results obtained supported the one-factor solution, as previously reported (χ^2^ (27) = 60.075; CFI = 0.990; TLI = 0.987; RMSEA = 0.056 [90% CI: 0.037–0.075], *p = 0*.274; SRMR = 0.076). As shown in Figure 1, all factor standardized factor loadings of the Spanish IGDS9-SF were considered high and statistically significant (λ > 0.55, *p* < 0.001), with all the residual correlations remaining close to zero. The unidimensional model is represented in Figure 1.

### 3.2. Convergent Validity, Construct Reliability, and Temporal Stability

Convergent validity is considered appropriate when the AVE of the latent variable is ≥0.50 and CR is ≥0.70, and there is no evidence of cross-loadings across the constructs [67,68]. The results of this analysis indicated an AVE value of 0.52 and CR of 0.91. Moreover, the Cronbach’s alpha coefficient was α = 0.90, whereas the Pearson correlation between test and retest scores was *r* = 0.89. Finally, the Omega reliability coefficient was also high (ω = 0.84). Taken together, these findings indicate that the Spanish IGDS9-SF presents with adequate construct validity, reliability, and temporal stability.

### 3.3. Associations between IGD and Other Relevant Variables

The descriptive statistics of the scores obtained with the tests administered in addition to the IGDS9-SF are shown in Table 1, as well as the correlations between these instruments and the IGDS9-SF total score. The IGDS9-SF was highly correlated with the IGD-20 Test score (*r* = 0.77, *p* < 0.01), moderately correlated with depression (*r* = 0.27, *p* < 0.01) and stress (*r* = 0.22, *p* < 0.01), and correlated to a lesser extent with anxiety (*r* = 0.17, *p* < 0.01). Additionally, in terms of personality traits, the largest correlations emerged between IGD and consciousness (*r* = −0.19, *p* < 0.01), followed by IGD and openness (*r* = 0.12, *p* < 0.05).

### 3.4. IRT Analysis of the Spanish IGDS9-SF

The BIC (BIC_PCM_ = 5430.68, BIC_GPCM_ = 5423.30, BIC_GRM_ = 5388.25) and AIC (AIC_PCM_ = 5288.09, AIC_GPCM_ = 5249.01, AIC_GRM_ = 5213.96) indices indicated that the GRM showed a better fit to the data. Table 2 shows all parameter estimates of the model and relevant item fit statistics. Statistically significant scores of S-χ^2^ indicate potential misfit to the model [69,70]. Accordingly, item fit was acceptable for all nine IGDS9-SF items. The item discrimination parameter (α) provides an estimate of the degree the item differentiates between individuals of varying trait levels (in *logit* scale).

According to Baker [71], the analysis of item parameters showed that the discrimination parameter of Item 8 (*escape*) was moderate (α_item8_ = 1.097), whereas it was high for Item 7 (*deception*) and Item 9 (*negative consequences*) (α_item7_ = 1.592 and α_item9_ = 1.631, respectively), and the remaining six items showed very high discrimination levels (α > 1.70). Each item threshold parameter or item difficulty (β) indicates the latent trait level (in *logit* scale) needed to have a 50% chance of selecting a particular response category or higher. The results suggested that no participant chose the response option “*often*” on Item 9 (*negative consequences*), preventing the item location threshold β_4_ from being estimated. Furthermore, threshold parameters were unevenly distributed across the trait range. Most items had difficulty parameters located in the upper-middle band of the latent trait (from −0.193 to 4.066), suggesting that most individuals are unlikely to endorse lower item response options.

**Figure 2:**
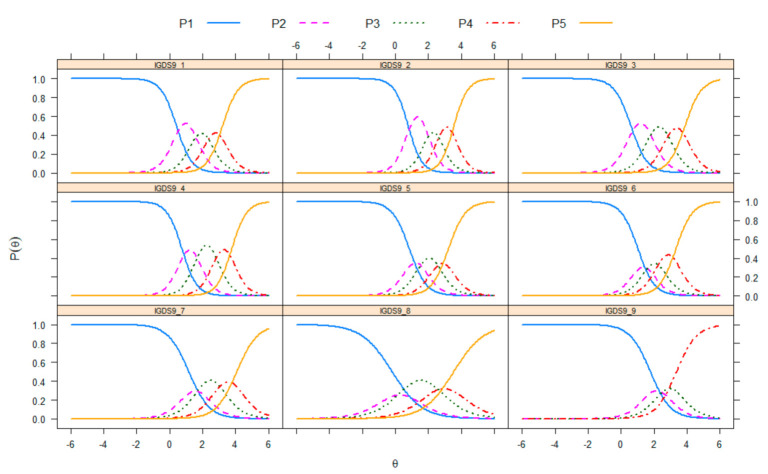
Category curves for the nine items of the Internet Gaming Disorder Scale-Short Form (IGDS9-SF).

Figure 2 presents the IGDS9-SF item category curves. There are multiple curves plotted per item (from the first response option P1 to the last response option P5), representing the probability of choosing a particular response option as a function of the latent trait. Although all items appeared to be monotonic, the response options of some items (i.e., Items 8 and 9) could potentially be reduced to three options, collapsing the option ‘*never’* with *‘rarely’* and ‘*often’* with ‘*very often’*.

**Figure 3:**
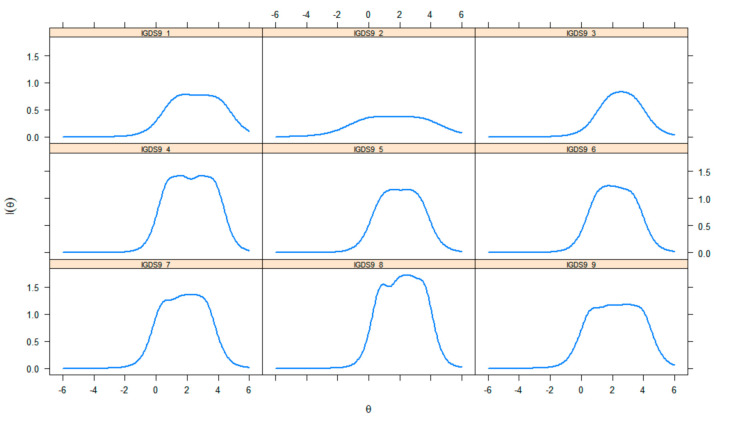
Internet Gaming Disorder Scale-Short Form (IGDS9-SF) item information curves.

**Figure 4:**
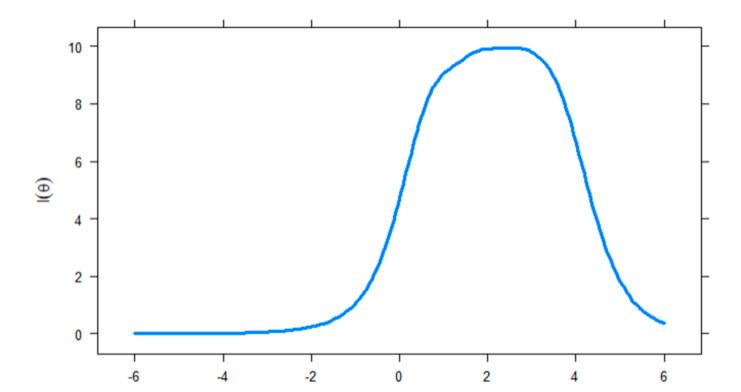
Test information curve of the Internet Gaming Disorder Scale-Short Form (IGDS9-SF).

The item information functions, which show the amount of information that each item explains as a function of their latent trait level, are presented in Figure 3. Consequently, most of the items were more informative at medium and high levels of the latent trait, especially Item 4 (*loss of control*), Item 7 (*deception*), and Item 8 (*escape*), whereas the least informative item was Item 2 (*withdrawal*). Similarly, Figure 4 contains the test information function, which indicates that the IGDS9-SF is less precise at the lower level of the trait (θ < 0) and becomes more informative when the trait level is between 0 and 4.

## 4. Discussion

The aim of the present study was to further analyze the psychometric properties of the Spanish IGDS9-SF beyond the CTT framework, so that its utility as a psychometric test for assessing IGD in Spanish samples can be ascertained. The results from the CFA provided additional support for the unidimensionality of the Spanish IGDS9-SF (with all standardized factor loadings being statistically significant and relatively high). The results also suggested that the Spanish IGDS9-SF presented with robust convergent validity, and in terms of reliability, the results indicated that the test was internally consistent and stable over a one-month period of time, similar to previous psychometric studies conducted with the IGDS9-SF [31].

The present study conducted a more detailed analysis of the psychometric properties of the Spanish IGDS9-SF by examining the characteristics of all nine items within the IRT framework. The polytomous IRT analyses suggested that the IGDS9-SF items presented with moderate to high discrimination parameters, and that they were more informative at higher levels of the latent trait being measured (i.e., IGD). In contrast to other studies [39], all of the items presented adequate fit to the IRT model, further indicating the suitability of the IGDS9-SF in assessing the nine IGD criteria as outlined in the DSM-5 [1], which overlap with the theoretical framework of the components model of addiction proposed by Griffiths [72].

Further evidence of validity in terms of expected relationships between IGD and other relevant variables was gathered to explore the direction and strength of associations between IGDS9-SF scores and gaming behaviors, personality traits, and psychiatric symptoms (depression, anxiety, and stress). As expected, participants’ IGDS9-SF scores were highly correlated with their IGD-20 Test scores, with a relatively similar strength to that reported in previous studies [4]. The results of this study also indicated statistically significant associations between IGDS9-SF total scores and symptoms of depression and stress as assessed with the DASS-21, further corroborating previous findings [73].

As for the personality traits examined, IGD and consciousness were inversely correlated. This is a finding that echoes those reported in the fields of internet addiction, gambling disorder, and substance addictions [22,74], all of which are related to the IGD construct [75]. In contrast to other studies [13], the correlation between IGD and neuroticism was weak. This result might be due to the relatively low reliability observed in the Mini-IPIP-PW neuroticism scale in the present study. Further research is necessary to deepen the assessment of the nomological network of constructs involved with IGD to further explore potential functional impairments and psychological features typically associated with IGD [27]. In this regard, additional evidence of convergent and discriminant validity should be explored in future studies.

The IGDS9-SF has been shown to be precise in assessing the high end of the latent trait (i.e., IGD). This is an important aspect for a clinical assessment tool because clinical cases of disordered gaming will often present with severe gaming-related problems and associated functional impairments [76]. This is highly relevant because IGD is a condition that needs to be clinically assessed effectively in order to facilitate clinical diagnosis and inform the development of preventative and intervention initiatives aimed at mitigating the harmful effects of disordered gaming on functioning. In sum, the results obtained support the notion that the IGDS9-SF is suitable to assess IGD at high levels, which is particularly relevant for a clinical diagnostic tool [39].

Although the results of the present study strongly support the robust psychometric properties of the Spanish IGDS9-SF, there are some potential limitations worth noting. Firstly, the sampling strategy used in the present study to recruit participants. Participants were self-selected, thus the results cannot be directly generalized to the general population. Secondly, because the sample size used in the test–retest reliability analysis was low, future studies examining the temporal stability of the IGDS6-SF scores should recruit larger samples to overcome the limitations in the present study. Thirdly, the clinical diagnosis of IGD using a gold standard was not possible, further preventing the authors from exploring the diagnostic accuracy of the IGDS9-SF in terms of its sensitivity and specificity among individuals clinically diagnosed with IGD. However, a recent study [47] suggested that using a cut-off of 32 points on the IGDS9-SF yields robust diagnostic capabilities (i.e., Youden’s index, 96.2%; diagnostic accuracy, 96.1%; sensitivity, 98%; specificity, 91.9%; negative predictive value, 100%; accuracy, 96.1%). Despite these potential limitations, the present study expands the previous work by Beranuy and colleagues [42] on the utility of the Spanish version of the IGDS9-SF, as it utilized a community-based sample across a wide age range (adolescents and adults). Another important contribution of the present study is that it provided necessary data on the IGDS9-SF’s temporal stability and incorporated IRT as a framework of analysis.

## 5. Conclusions

The major contribution of the present study is that it is one of the few studies to analyze item and test performance of the IGDS9-SF utilizing the IRT framework, being the first in the Spanish context, which contains a large portion of gamers globally. Overall, the results indicated that the IGDS9-SF factor structure was unidimensional, test scores were consistent and stable over time, with items ranging from being moderate to strong discriminators of the IGD trait. Furthermore, the IGDS9-SF was more precise at the higher severity levels of the IGD trait, which makes this tool particularly promising for clinical and epidemiological studies in Spanish-speaking contexts.

## Figures and Tables

**Table 1:** Descriptive statistics of the Internet Gaming Disorder Test-20 (IGD-20 Test); the Mini International Personality Item Pool-Five-Factor Model-Positively Worded (Mini-IPIP-PW); and the Depression, Anxiety, and Stress Scales (DASS-21), as well as their correlations with the Internet Gaming Disorder Scale-Short Form (IGDS9-SF).

Instruments/Dimensions	Mean	SD	α	ω	*r*
IGD-20 Test	35.18	12.03	0.95	0.77	0.77 **
Mini-IPIP-PW					
Neuroticism	11.23	3.36	0.72	0.69	0.01
Extraversion	10.80	3.84	0.79	0.57	−0.05
Openness	14.06	3.77	0.85	0.89	0.12 *
Agreeableness	15.20	3.56	0.90	0.85	−0.04
Consciousness	12.85	3.61	0.85	0.84	−0.19 **
DASS-21					
Anxiety	4.26	4.40	0.89	0.75	0.17 **
Depression	5.57	5.17	0.92	0.89	0.27 **
Stress	7.09	4.92	0.87	0.62	0.22 **

Note: SD: standard deviation; α = Cronbach’s alpha coefficient; ω = Omega coefficient, * *p* < 0.05, ** *p* < 0.01.

**Table 2:** Item statistics for the graded response model (GRM) across all items of the Spanish Internet Gaming Disorder Scale-Short Form (IDGS9-SF).

IGDS9-SF Items	α	β_1_	β_2_	β_3_	β_4_	S-χ^2^	df	*p*
Item 1	2.127	0.413	1.508	2.354	3.206	23.210	19	0.228
Item 2	2.406	0.781	1.910	2.674	3.550	21.436	17	0.207
Item 3	2.010	0.600	1.770	2.839	3.875	23.010	17	0.149
Item 4	2.218	0.747	1.706	2.795	3.763	21.759	18	0.243
Item 5	1.943	0.851	1.603	2.481	3.217	12.198	21	0.934
Item 6	1.987	1.025	1.662	2.382	3.319	23.570	21	0.314
Item 7	1.592	1.166	1.929	3.023	4.066	10.645	21	0.969
Item 8	1.097	−0.193	0.754	2.330	3.534	34.161	28	0.196
Item 9	1.631	1.782	2.541	3.356	-	24.169	17	0.115

Note: α: discrimination parameter; β: difficulty parameter; S-χ^2^: generalized chi-square statistic [69,70], df: degrees of freedom, *p*: *p*-value.

## Appendix A

**Table A1:** Spanish version of the IGDS9-SF (Internet Gaming Disorder Scale-Short Form).

**Instrucciones:** Las siguientes preguntas se refieren a tu actividad de juego con videojuegos durante el último año (es decir, los últimos 12 meses). Por actividad de juego con videojuegos nos referimos a cualquier actividad de juego llevada a cabo desde un ordenador (de sobremesa o portátil) o desde una consola o cualquier otro dispositivo (por ejemplo, móvil, tableta, etc.), tanto de forma *online* (con conexión a internet) como *offline* (sin conexión a internet).					
**Preguntas**	**Nunca**	**Raramente**	**A veces**	**A menudo**	**Muy a menudo**
¿Te sientes preocupado/a con tu conducta de juego? (Por ejemplo: ¿piensas en tu actividad de juego anterior o anticipas la próxima sesión de juego? ¿Crees que jugar se ha convertido en la actividad principal de tu vida diaria?)					
2. ¿Te sientes más irritable, ansioso/a o incluso triste cuando intentas reducir o parar tu actividad de juego con videojuegos?					
3. ¿Sientes la necesidad de pasar cada vez más tiempo jugando a videojuegos para lograr satisfacción o placer?					
4. ¿Fracasas sistemáticamente cuando intentas controlar o cesar tu actividad de juego con videojuegos?					
5. ¿Has perdido interés por tus anteriores aficiones o actividades de entretenimiento como consecuencia de tu implicación con los videojuegos?					
6. ¿Has continuado jugando a videojuegos aun sabiendo que te estaba causando problemas con otras personas?					
7. ¿Has engañado a algún miembro de tu familia, terapeuta u otra persona sobre la cantidad de tiempo que dedicas a los videojuegos?					
8. ¿Juegas para escapar o aliviar temporalmente un estado de ánimo negativo (por ejemplo, impotencia, culpa, ansiedad)?					
9. ¿Has puesto en riesgo o perdido una relación importante, un trabajo o una oportunidad académica o profesional debido a los videojuegos?					
